# Identification and validation of hub genes and pathways associated with mitochondrial dysfunction in hypertrophy of ligamentum flavum

**DOI:** 10.3389/fgene.2023.1117416

**Published:** 2023-05-10

**Authors:** Yifei Gu, Wenchao Yu, Min Qi, Jinquan Hu, Qianmei Jin, Xinwei Wang, Chen Wang, Yu Chen, Wen Yuan

**Affiliations:** ^1^ Department of Orthopaedics, Changzheng Hospital, Naval Medical University, Shanghai, China; ^2^ Department of Rheumatology and Immunology, Changzheng Hospital, Naval Medical University, Shanghai, China

**Keywords:** mitochondrial dysfunction, hypertrophy of ligamentum flavum, GEO, differentially expressed genes, bioinformatics analysis

## Abstract

**Background:** Lumbar spinal stenosis which can lead to irreversible neurologic damage and functional disability, is characterized by hypertrophy of ligamentum flavum (HLF). Recent studies have indicated that mitochondrial dysfunction may contribute to the development of HLF. However, the underlying mechanism is still unclear.

**Methods**: The dataset GSE113212 was obtained from the Gene Expression Omnibus database, and the differentially expressed genes were identified. The intersection of DEGs and mitochondrial dysfunction-related genes were identified as mitochondrial dysfunction-related DEGs. Gene Ontology analysis, Kyoto Encyclopedia of Genes and Genomes (KEGG) analysis, and Gene Set Enrichment Analysis were performed. Protein-protein interaction network was constructed, and miRNAs and transcriptional factors of the hub genes were predicted *via* the miRNet database. Small molecule drugs targeted to these hub genes were predicted *via* PubChem. Immune infiltration analysis was performed to evaluate the infiltration level of immune cells and their correlation with the hub genes. In final, we measured the mitochondrial function and oxidative stress *in vitro* and verified the expression of hub genes by qPCR experiments.

**Results**: In total, 43 genes were identified as MDRDEGs. These genes were mainly involved in cellular oxidation, catabolic processes, and the integrity of mitochondrial structure and function. The top hub genes were screened, including LONP1, TK2, SCO2, DBT, TFAM, MFN2. The most significant enriched pathways include cytokine-cytokine receptor interaction, focal adhesion, etc. Besides, SP1, PPARGC1A, YY1, MYC, PPARG, and STAT1 were predicted transcriptional factors of these hub genes. Additionally, increased immune infiltration was demonstrated in HLF, with a close correlation between hub genes and immune cells found. The mitochondrial dysfunction and the expression of hub genes were validated by evaluation of mitochondrial DNA, oxidative stress markers and quantitative real-time PCR.

**Conclusion:** This study applied the integrative bioinformatics analysis and revealed the mitochondrial dysfunction-related key genes, regulatory pathways, TFs, miRNAs, and small molecules underlying the development of HLF, which improved the understanding of molecular mechanisms and the development of novel therapeutic targets for HLF.

## 1 Instruction

Lumbar spinal stenosis (LSS) is the most common spinal disorder in the elderly population worldwide with an approximate prevalence of 11% in adults and 20% in people older than 60 (Katz et al., 2022). The pathological mechanisms of LSS are very diverse, while hypertrophy of ligamentum flavum (HLF) has been generally accepted as one of the key contributors of LSS (Walter and O'Toole, 2022). HLF can cause compression of the nerve roots and cauda equina, leading to symptoms including intermittent claudication, leg pain, paresthesia and motor dysfunction in the lower extremities, and even urinary and/or fecal incontinence. The severity of HLF could increase with age, and if not properly treated, it may lead to irreversible and disabling consequences (Shamji et al., 2015). As an age-related degeneration, the major pathological change of HLF is fibrosis, which is manifested by the loss of elastic fibers and the increase of collagen fibers with the disordered arrangement in the ligament tissue (Sun et al., 2020). However, the mechanisms underlying HLF are still poorly understood.

Mitochondria are double membrane-enclosed organelles that play an essential role in energy metabolism and cellular processes including cell proliferation, apoptosis, and inflammatory responses (Kudryavtseva et al., 2016), while mitochondrial dysfunction has been proven to play an important role in aging and degenerative processes (Kasapoglu and Seli, 2020). Several studies have suggested that mitochondrial dysfunction was associated with various degenerative diseases of the musculoskeletal system including osteoarthritis chondrocytes (Liu et al., 2019) and intervertebral disc degeneration (Saberi et al., 2021). Liu et al. (2019) revealed that mitochondrial function was impaired in osteoarthritis chondrocytes, resulting in increased chondrocyte apoptosis and decreased type ii collagen secretion. Saberi et al. (2021) suggested that mitochondrial dysfunction results in oxidative stress, cell death, and premature cell senescence, which are all implicated in intervertebral disc degeneration. Recently, Chuang et al. (2020) demonstrated that oxidative stress induces inflammatory responses and the subsequent fibrotic processes in ligamentum flavum and revealed that mitochondrial dysfunction may contribute as an etiological factor of HLF. Therefore, the exploration of mitochondrial dysfunction in HLF has potential value for clinical diagnosis, prognostic assessment, and targeted therapy, but its detailed mechanism and molecular targets remain unclear.

Bioinformatics analysis provides a global perspective for understanding the molecular mechanisms of disease, exploring relevant novel biomarkers for diagnosis and prognosis, and studying possible therapeutic targets. Via the method of enrichment and protein-protein interaction (PPI) network analyses, Pan et al. (2021) investigated the potential key genes and signaling pathways related to cell proliferation in HLF and revealed that AdAM10 promoted the proliferation of ligamentum flavum cells by activating the PI3K/AKT pathway. However, to date, there is no relevant report describing bioinformatics analysis on mitochondrial dysfunction in the process of HLF.

Therefore, this study aimed to find out genes and pathways related to mitochondrial dysfunction in HLF from the point of view of bioinformatics analysis and provide a theoretical basis for clinical diagnosis and targeted therapy.

## 2 Methods and materials

### 2.1 Microarray data and preparation

The gene expression profile of dataset GSE113212 was obtained from the Gene Expression Omnibus (GEO) database (http://www.ncbi.nlm.nih.gov/geo/), National Center for Biotechnology Information (NCBI). This data set was based on the GPL17077 platform and contained a total of 8 samples, including 4 hypertrophic ligamentum flavum samples from the elderly individuals and 4 non-hypertrophic samples from the young individuals (Table 1). We performed standard procedures of quality control on the dataset *via* the R package array QualityMetrics (Kauffmann et al., 2009), and log2 normalization using the R package limma (Ritchie et al., 2015). In addition, principal component analysis (PCA) was performed using the R packages FactoMineR (Luo et al., 2014) and factoextra (Kassambara and Mundt, 2020). Differentially expressed genes (DEG) are derived from differential analysis by the R package limma and visualized using heatmaps and volcano plots. For identifying the significant DEGs, we set the |log (fold-change) | > 0 and *p* < 0.05 as the cutoff.

**TABLE 1:** Results of GO and KEGG analysis.

Ontology categories	Description	*p*-value
BP	organic acid catabolic process	2.20e-11
BP	carboxylic acid catabolic process	2.20e-11
BP	mitochondrial respiratory chain complex assembly	2.11e-09
BP	small molecule catabolic process	3.61e-09
BP	fatty acid beta-oxidation	1.45e-08
BP	oxidative phosphorylation	3.76e-08
BP	fatty acid oxidation	9.92e-08
BP	mitochondrial transport	1.07e-07
BP	lipid oxidation	1.12e-07
BP	branched-chain amino acid metabolic process	1.42e-07
CC	mitochondrial matrix	7.74e-25
CC	mitochondrial inner membrane	1.55e-21
CC	mitochondrial protein complex	4.28e-09
CC	mitochondrial membrane part	2.99e-08
CC	nucleoid	3.51e-08
CC	mitochondrial nucleoid	3.51e-08
CC	oxidoreductase complex	4.38e-06
CC	intrinsic component of mitochondrial membrane	2.02e-05
CC	mitochondrial respiratory chain	4.00e-05
CC	respiratory chain	6.60e-05
MF	coenzyme binding	4.27e-07
MF	oxidoreductase activity, acting on NAD(P)H	1.30e-04
MF	NADH dehydrogenase activity	1.89e-04
MF	NADH dehydrogenase (ubiquinone) activity	1.89e-04
MF	NADH dehydrogenase (quinone) activity	1.89e-04
MF	oxidoreductase activity, acting on the CH-CH group of donors	3.76e-04
MF	oxidoreductase activity, acting on NAD(P)H, quinone or similar compound as acceptor	4.15e-04
MF	adenine nucleotide transmembrane transporter activity	5.15e-04
MF	purine ribonucleotide transmembrane transporter activity	5.15e-04
MF	purine nucleotide transmembrane transporter activity	5.93e-04
KEGG		
ID	Description	*p*-value
hsa00280	Valine, leucine and isoleucine degradation	1.37e-08
hsa01212	Fatty acid metabolism	4.64e-05
hsa00640	Propanoate metabolism	2.31e-04
hsa00071	Fatty acid degradation	4.99e-04
hsa04146	Peroxisome	0.003
hsa00062	Fatty acid elongation	0.004
hsa00650	Butanoate metabolism	0.004
hsa00630	Glyoxylate and dicarboxylate metabolism	0.005

### 2.2 Identification of mitochondrial dysfunction-related differentially expressed genes (MDRDEGs)

To analyze the expression difference of mitochondrial dysfunction-related genes in all samples, the list of 1,136 genes encoding proteins with strong support of mitochondrial localization, sub-mitochondrial compartment and pathway annotations were downloaded from the MitoCarta3.0 database (http://www.broadinstitute.org/mitocarta) (Rath et al., 2021). From them, 446 mitochondrial dysfunction-related genes were obtained by searching for the keyword “mitochondrial dysfunction” in the GeneCards database (https://www.genecards.org/) (Safran et al., 2010). Then the intersection of these selected mitochondrial dysfunction-related genes (MD genes) and the DEGs from dataset GSE113212 was identified as MDRDEGs.

### 2.3 Functional enrichment analysis

To systematically explore the underlying biological functions of identified MDRDEGs in HLF, Gene Ontology (GO) enrichment analysis and Kyoto Encyclopedia of Genes and Genomes (KEGG) enrichment analysis were performed *via* the R package clusterProfiler (Yu et al., 2012). GO analysis involved three categories: Biological process, cellular component, and molecular function. Gene Set Enrichment Analysis (GSEA) was performed using the R package clusterProfiler.

### 2.4 Correlation Analysis

The R package RCircos (Zhang et al., 2013) is used to map the location of genes in chromosomes. The chromosome data is provided by the R package itself, and the location information of genes on chromosomes is downloaded from the ENSEMBL database (Yates et al., 2020). Pearson correlation between the expression of identified MDRDEGs in elderly individuals was performed.

### 2.5 Protein-protein interaction network construction

The protein-protein interaction (PPI) network was constructed based on the Search Tool for the Retrieval of Interacting Genes (STRING) database with the MDRDEGs as input. Medium confidence (interaction score > 0.4) was chosen as a threshold and all positive interactions were included. The results were visualized using Cytoscape software (Shannon et al., 2003). The hub genes were filtered from the whole PPI network using CytoHubba (Chin et al., 2014), a plugin of Cytoscape software and calculated by the Degree method. Twenty genes in the maximum correlation criterion (MCC) were chosen by the CytoHubba plugin and sequentially ordered.

### 2.6 Construction of Genes-miRNAs and genes-transcription factors (TFs) network

To further understand the regulation of these hub genes, we constructed genes-miRNAs and genes-transcription factors (TFs) regulatory network based on the miRNet database (Chang et al., 2020) predicted the miRNAs and TFs of the hub genes respectively.

### 2.7 Immune infiltration analysis

To evaluate the infiltration level of immune cells, and the content of 22 different types of immune cells in each sample was calculated based on the LM22 background gene set provided by CIBERSORT (Chen et al., 2018). To further reveal the correlation between the hub genes and the infiltration level of immune cells, a correlation scatter plot for the gene-immune cell pair with a significant correlation with the fitting curve was drawn.

### 2.8 LF sample collection and cell isolation

To further verify the selected hub MDRDEGs and related pathways, ligamentum flavum (LF) samples were collected from 30 patients (12 females and 18 males) who underwent posterior lumbar decompression surgery with removal of LF tissue from June 2021 to January 2022. The process of biosample collection followed the ethical approval of the Naval Medical University. For the HLF group, 15 LF specimens were collected from LSS patients with LF hypertrophy, and for the LDH group, 15 specimens were harvested from patients with uncomplicated lumbar disc herniation as control. The thickness of LF was measured on T2-weighted magnetic resonance imaging (MRI) and LF hypertrophy was defined as LF thickness > 4 mm according to the previous study (Zheng et al., 2020). Table 2 summarized the demographic variable of the cases included in different groups. Extensive or partial laminectomy with LF resection was performed in all patients during the operation. The resected ligamentum flavum was rinsed in 4°C physiological saline and then sent for examination immediately.

**TABLE 2:** Patient demographics for verification.

	LDH	HLF	*p*-value
Number of Cases	15	15	
Sex (female/male)	5/10	6/9	0.705
Age (years)	32.73 ± 10.19	69.00 ± 4.84	**< 0.001**
LF thickness (mm)	2.57 ± 0.52	5.35 ± 0.77	**< 0.001**

LDH: lumbar disc herniation; HLF: hypertrophy of the ligamentum flavum.

The bold values indicates statistically significant differences.

Ligamentum flavum cells were isolated as described previously (Specchia et al., 2001; Zheng et al., 2020). In brief, LF tissue was washed by PBS 3 times, cut into small pieces measuring around 0.5 mm^3^, and digested for one hour with 0.2% type I collagenase (Gibco), The digested fragments were then rinsed in DMEM (Gibco), supplemented with 10% FBS (Glpbio, United States), and 100 U/mL penicillin. Cells after the third passage were used for experiments.

### 2.9 Evaluation of mitochondrial DNA (mtDNA) and oxidative stress markers

LF samples were homogenised to isolate total DNA. real-time quantitative PCR was performed on the mitochondrial gene cytochrome c oxidase subunit 1 (COX1) and the nuclear DNA product (b-actin). The relative mtDNA copy number was calculated by subtracting the cycle of the nuclear gene from the cycle of the mitochondrial gene.

The level of ROS in LF cells was assessed by a C11-BODIPY probe assay kit (Invitrogen) according to the instructions. 1 × 10^4^ LF cells were seeded in 96-well plates and cultured for 30 min with a 2 μM C11-BODIPY probe, and the number of reactive oxygen species (ROS) was measured using a flow cytometer. The malondialdehyde (MDA) and glutathione (GSH) content in tissue homogenates and cell lysis were analyzed by a lipid peroxidation kit (Sigma, MAK085) and Glutathione Assay Kit (Sigma, CS0260) by the standard protocol.

### 2.10 Quantitative real-time PCR (qPCR)

About three cubic meters of ligamentum flavum tissue were homogenized in 800 μL of Trizol. Total RNA was isolated by Trizol reagent (Invitrogen, United States) according to the manufacturer’s instructions, and further reverse transcribed by using the iScript cDNA Synthesis kit (bio-rad). Real-time PCR and analysis were performed as previously described (Xu et al., 2016a). The fold changes of target genes were analyzed by the 2^−ΔΔCT^ method and 18s was used as an internal control.

### 2.11 Statistical analysis

The Wilcoxon rank sum test was used to compare the differences between groups. All statistical analyses and visualizations were performed *via* R software (version 4.0.2). *p* < 0.05 was considered to be significantly different, and all statistical tests were two-sided.

## 3 Results

### 3.1 Data preparation and DEGs

**FIGURE 1:**
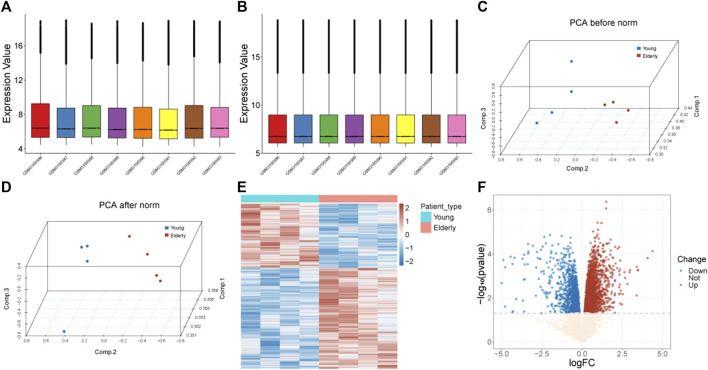
Data preprocessing and identification of DEGs. **(A)** Box plots of gene expression before normalization. **(B)** Box plots of gene expression after normalization. **(C)** PCA analysis before normalization. The red nodes represent the elderly group and the blue nodes represent the control group. **(D)** PCA analysis after normalization. The red nodes represent the elderly group and the blue nodes represent the control group. **(E)** Heatmap of identified DEGs. The red color represents genes with high expression, and the blue color represents genes with low expression. **(F)** Volcano plot of DEGs. The blue dots represent downregulated genes, the red dots represent upregulated genes, and the light-colored dots represent genes with no significant change in expression. Abbreviation: DEGs: differentially expressed genes; PCA: Principal Component Analysis.

The gene expression profile of the GS113212 was normalized and shown in Figures 1A–D. A total of 3,742 genes were identified as DEGs, including1,457 downregulated ones and 2,285 upregulated ones (Figures 1E, F).

### 3.2 Identification of MDRDEGs and functional enrichment analysis

**FIGURE 2:**
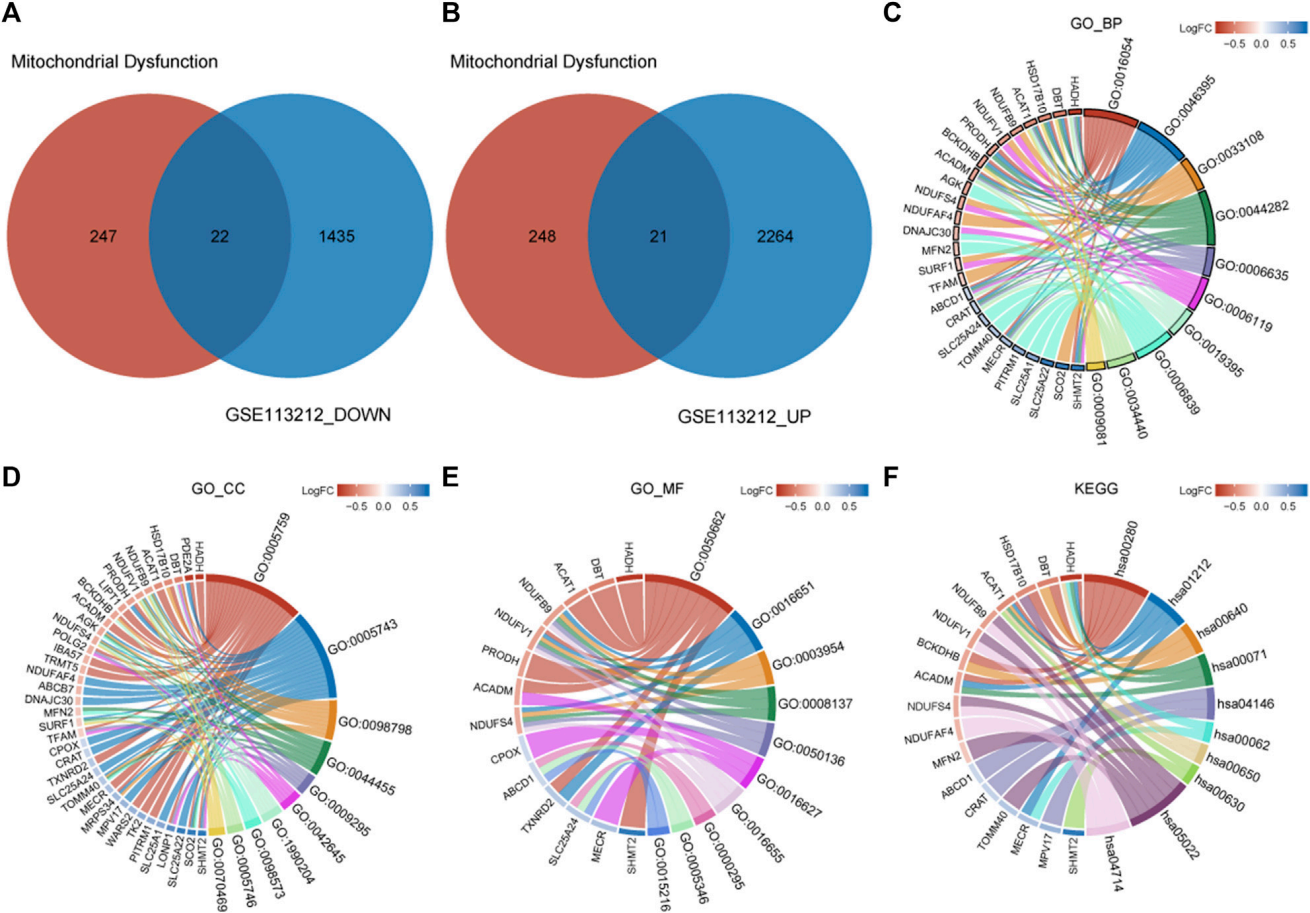
Identification of MDRDEGs and enrichment analysis. **(A)** Venn diagram of downregulated DEGs (blue) *versus* downregulated MDRGs (red). **(B)** Venn diagram of upregulated DEGs (blue) *versus* upregulated MDRGs (red). **(C)** Chord diagram of top 10 GO BP enrichment results. **(D)** Chord diagram of top 10 GO CC enrichment results. **(E)** Chord diagram of top 10 GO MF enrichment results. **(F)** Chord diagram of top 10 KEGG enrichment results. Abbreviation: DEGs: mitochondrial dysfunction-related genes; MDRDEGs: mitochondrial dysfunction-related differentially expressed genes; MDRGs: mitochondrial dysfunction-related genes; BP: biological process; CC: cellular components; MF: molecular function; GO: Gene Ontology; KEGG: Kyoto Encyclopedia of Genes and Genomes.

A total of 43 genes were derived from the intersection of mitochondrial dysfunction-related genes with the DEGs as MDRDEGs. There were 22 downregulated genes and 21 upregulated genes (Figure 2). The most significant enrichment terms for GO were organic acid catabolic process, carboxylic acid catabolic process, mitochondrial respiratory chain complex assembly (biological processes), mitochondrial matrix, mitochondrial inner membrane, mitochondrial protein complex (cellular components), coenzyme binding, oxidoreductase activity, and NADH dehydrogenase activity (molecular functions) (Figure 2; Table 1). KEGG pathway analysis demonstrated that the MDRDEGs were mainly enriched in valine, leucine, and isoleucine degradation, fatty acid metabolism, propanoate metabolism, and fatty acid degradation (Figure 2; Table 1).

**FIGURE 3:**
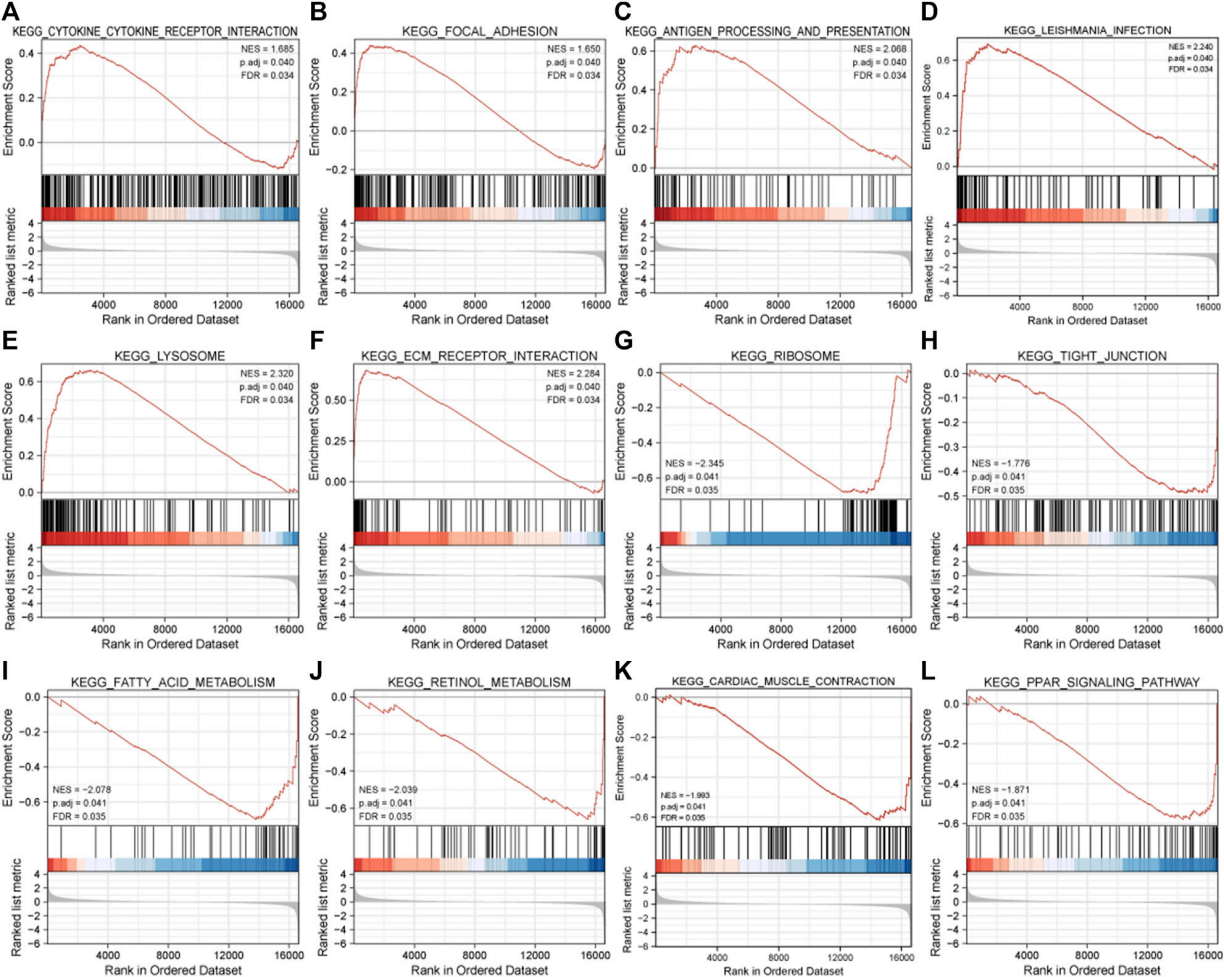
The twelve enrichment plots from the GSEA results. The gene sets of **(A)** cytokine-cytokine receptor interaction, **(B)** focal adhesion, **(C)** antigen processing and presentation, **(D)** leishmania infection, **(E)** lysosome, **(F)** ECM receptor interaction, **(G)** ribosome, **(H)** tight junction, **(I)** fatty acid metabolism, **(J)** retinol metabolism, **(K)** cardiac muscle contraction, (L) PPAR signaling pathway were significantly enriched in HLF samples based on GSE113212. Abbreviation: GSEA: Gene Set Enrichment Analysis, ECM: extracellular matrix, PPAR: peroxisome proliferator-activated receptors.

The result of GSEA showed that most of the MDRDEGs were involved in cytokine-cytokine receptor interaction, focal adhesion, antigen processing and presentation, lysosome, ECM receptor interaction, ribosome, tight junction, fatty acid metabolism, retinol metabolism, PPAR signal pathway (Figure 3; Table 3).

**TABLE 3:** Results of GSEA analysis.

Description	NES	p.adjust
KEGG_CYTOKINE_CYTOKINE_RECEPTOR_INTERACTION	1.685329	0.0395
KEGG_FOCAL_ADHESION	1.650383	0.0395
KEGG_ANTIGEN_PROCESSING_AND_PRESENTATION	2.068126	0.0395
KEGG_LEISHMANIA_INFECTION	2.239501	0.0395
KEGG_LYSOSOME	2.3195	0.0395
KEGG_ECM_RECEPTOR_INTERACTION	2.284356	0.0395
KEGG_RIBOSOME	−2.34489	0.040737
KEGG_TIGHT_JUNCTION	−1.77631	0.040737
KEGG_FATTY_ACID_METABOLISM	−2.07839	0.040737
KEGG_RETINOL_METABOLISM	−2.03898	0.040737
KEGG_CARDIAC_MUSCLE_CONTRACTION	−1.99273	0.040737
KEGG_PPAR_SIGNALING_PATHWAY	−1.87083	0.040737

### 3.3 Genetic landscape of MDRDEGs and correlation Analysis

**FIGURE 4:**
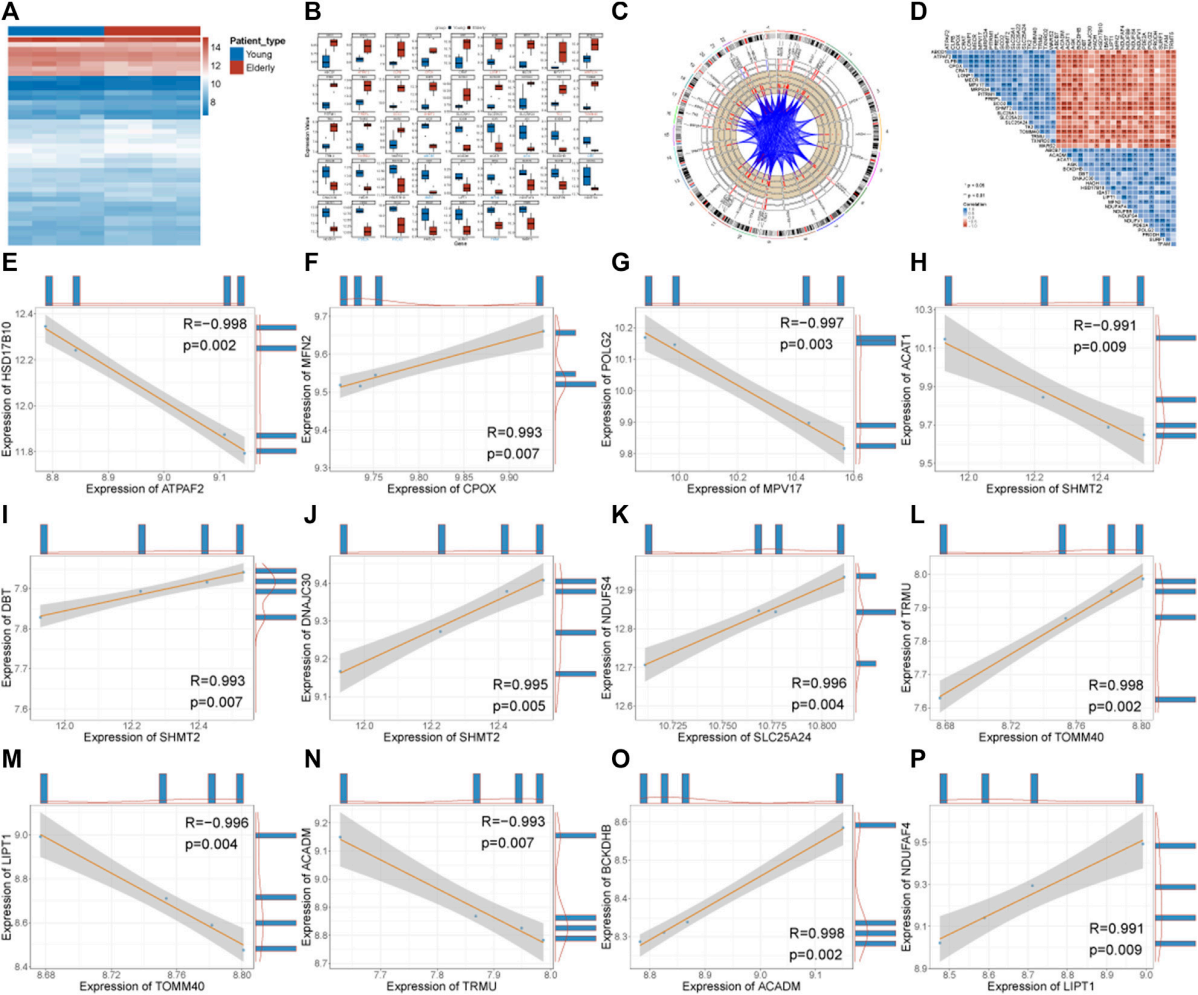
Panorama of MDRDEGs and Correlation Analysis. **(A)** Heatmap of MDRDEGs. The red color represents genes with high expression, and the blue color represents genes with low expression value. **(B)** Boxplots of MDRDEGs. The genes with red color present those with significantly higher expression in the elderly group, and genes with blue color present those with significantly lower expression in the elderly group. **(C)** Genetic landscape of MDRDEGs. **(D)** Heatmap of correlations between MDRDEGs. **(E-P)** Scatter plot of correlations between MDRDEGs. **A**bbreviation: MDRDEGs: mitochondrial dysfunction-related differentially expressed genes.

The expression of MDRDEGs was shown with a heatmap (Figure 4A). The expression differences of all MDRDEGs in young and elderly samples were visualized with grouped box plots (Figure 4B). Compared with the young group, a higher expression of ATPAF2, CLPB, CPOX, LONP1, MRPS34, PREPL, SCO2, SHMT2, TK2, TOMM40 and TXNRD2, and a lower expression of ABCB7, AGK, DBT, IBA57, MFN2, PDE2A, POLG2 and TFAM were observed in the elderly group. The locations of these genes were presented in the genetic landscape (Figure 4C).

Correlations between the expression of MDRDEGs were calculated and presented as a heat map (Figure 4D). Among them, a total of 12 gene pairs met the threshold of statistical significance with an association greater than 0.9 and *p* < 0.01. They were ATPAF2-HSD17B10, CPOX-MFN2, MPV17-POLG2, SHMT2-ACAT1, SHMT2-DBT, SHMT2-DNAJC30, SLC25A24-NDUFS4, TOMM40-TRMU, TOMM40-LIPT1, TRMU-ACADM, ACADM-BCKDHB, LIPT1-NDUFAF4 (Figures 4E–P). There were 7 pairs with positive correlations and 5 pairs with negative correlations.

### 3.4 Construction of PPI network

**FIGURE 5:**
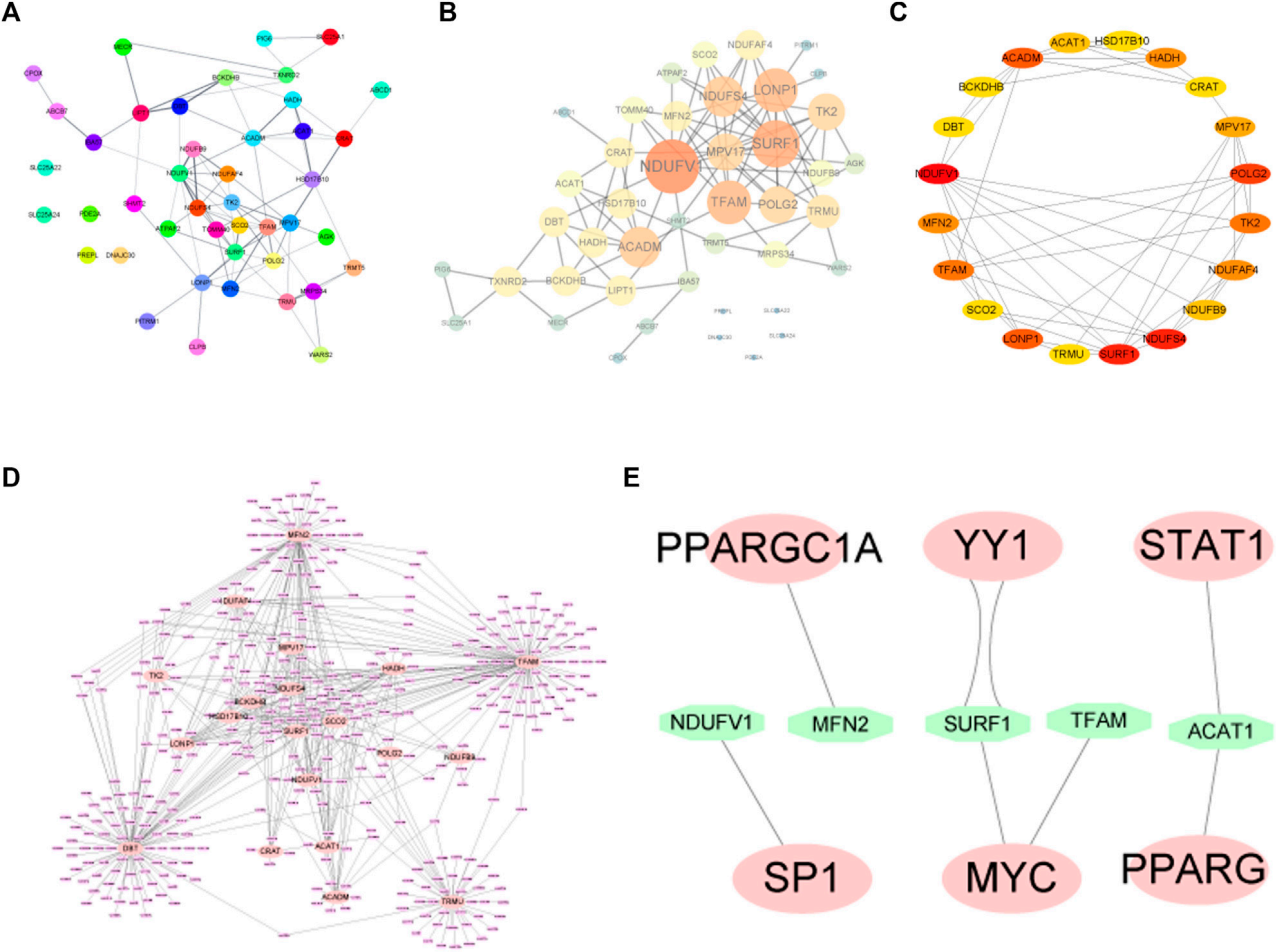
**(A,B)** PPI network diagram of MDRDEGs. **(C)** For the 20 hub genes extracted from the PPI network, the darker the red color, the higher the degree of the nodes in the original network. **(D)** The miRNA prediction network of hub genes. The pink nodes represent the mRNAs of hub genes and the purple nodes represent miRNAs. **(E)** Hub genes-transcription factor prediction network. The green nodes represent transcription factors. Abbreviation: PPI: Protein-protein interaction.

To determine the relationships among the mitochondrial dysfunction-related genes, a PPI network was constructed using the STRING database and then visualized using Cytoscape software (Figures 5A, B). According to the degree of the nodes, the top 20 genes including NDUFV1, SURF1, NDUFS4, POLG2, LONP1, ACADM, TK2, HADH, MPV17, NDUFAF4, TFAM, MFN2, NDUFB9, ACAT1, CRAT, HSD17B10, DBT, BCKDHB, SCO2, and TRMU were identified as hub genes (Figure 5C).

### 3.5 The Gene-miRNA and Gene-TF regulating network analysis

The transcription factors (TFs) and miRNAs of these hub genes were predicted using the miRNet database for a comprehensive analysis of their genetic background and regulatory network.

The gene-miRNA network consists of 20 hub genes and miRNAs (Figure 5D). The four interactive hub genes that most miRNA would target were MFN2, TFAM, TRMU, and DBT. Furthermore, the network showed that some of the miRNAs have multiple target hub genes.

A total of 8 pairs of gene-TF interactions among 5 hub genes including NDUFV1, MFN2, SURF1, TFAM, and ACAT1, and 6 TFs including SP1, PPARGC1A, YY1, MYC, STAT1, and PPARG were identified (Figure 5E).

### 3.6 Immune infiltration analysis

**FIGURE 6:**
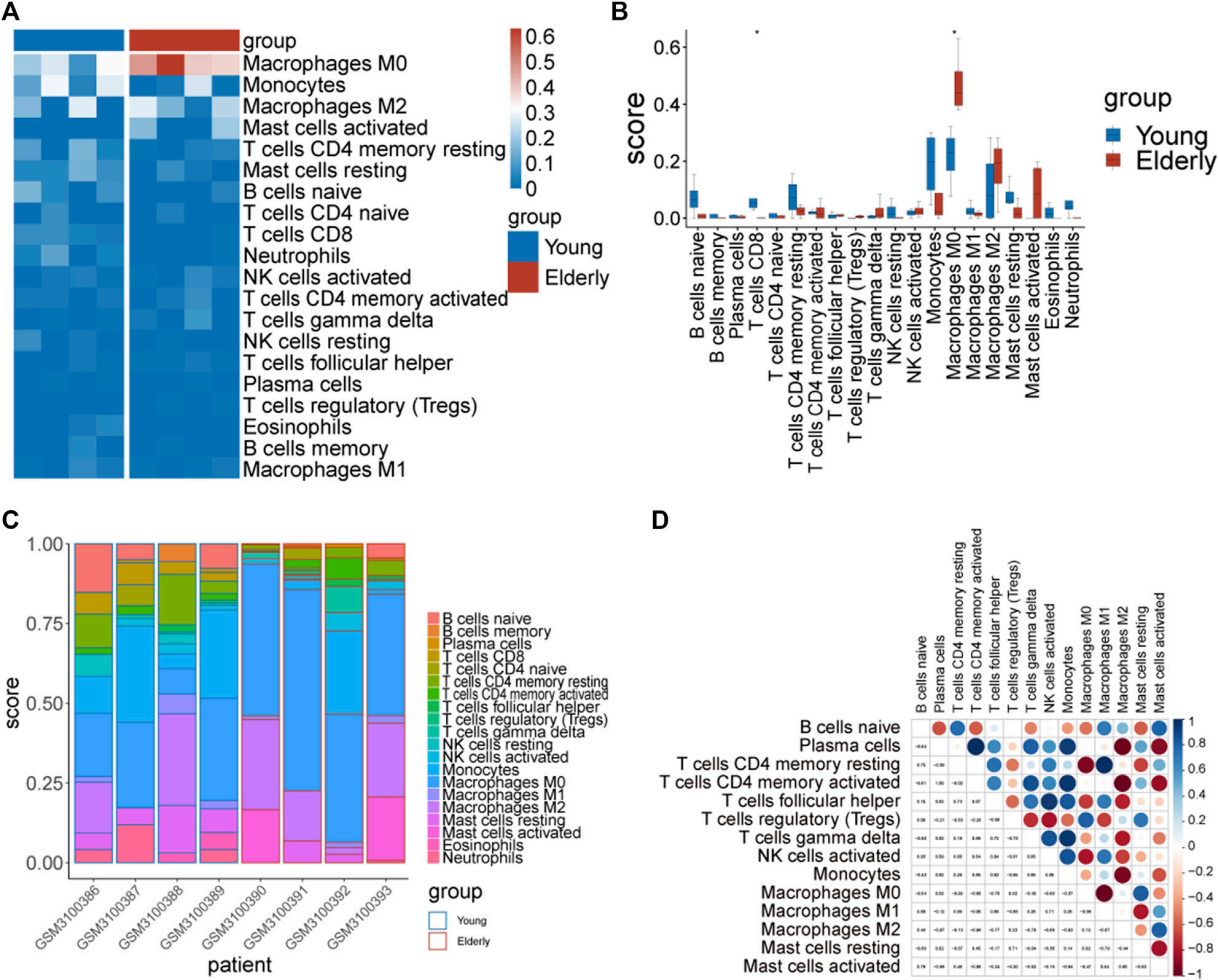
The immune infiltration assessment by Cibersort. **(A)** Heatmap of the immune score. The blue color represents immune cells with a low immune infiltration score and the red color represents immune cells with a high immune infiltration score. **(B)** Differences in the immune infiltration score of immune cells between the two groups. **(C)** Distribution of immune cells in different samples. **(D)** Correlation diagram of immune cells in elderly group samples.

From the enrichment analysis results, we found that there were significant differences in the immune process between the two groups of samples. Therefore, immune infiltration levels of 22 types of immune cells in the two groups of samples were analyzed. Significant differences between groups in CD8^+^ T cells and M0 macrophages were observed (Figures 6A–C).

Correlations between immune cells were analyzed separately in the two groups of samples. The results showed that there were strong correlations between the infiltration levels of immune cells in the elderly group (Figure 6D).

We further analyzed the correlation between hub genes and immune cells, and the results showed that ACADM, BCKDHB, HSD17B10, MFN2, and NDUFAF4 were positively correlated with B cells naïve, resting memory CD4^+^ T cells, regulatory T cells (Tregs) and M1 Macrophages, respectively, while the LONP1 gene was negatively correlated with resting memory CD4^+^ T.

### 3.7 Verification of the mitochondrial function

**FIGURE 7:**
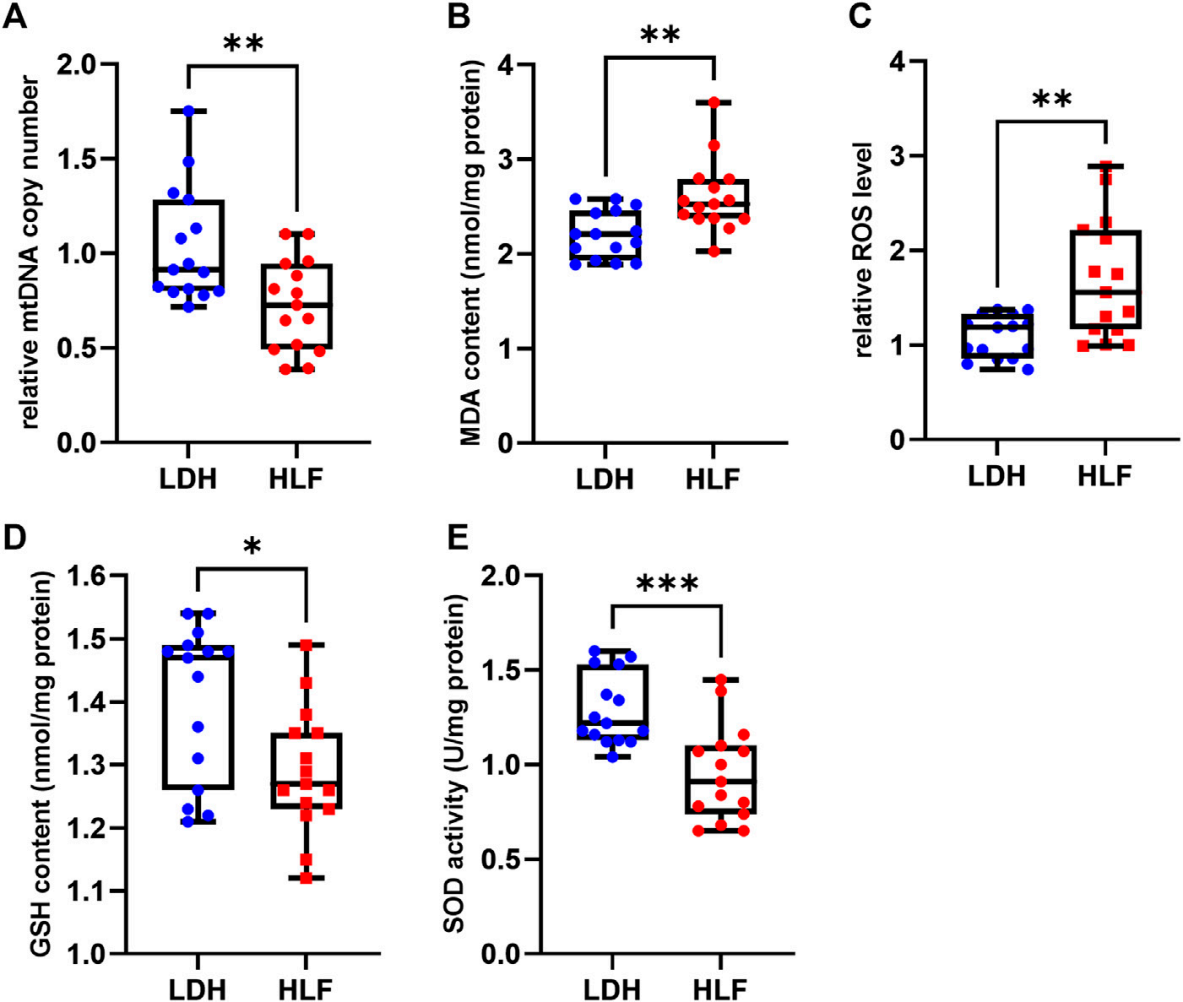
Measurements of mitochondrial function and oxidative stress. **(A)** The relative mtDNA copy number was significantly higher in the HLF group. **(B)** The ROS levels and **(C)** malondialdehyde (MDA) were higher in the HLF group compared with the LDH group. **(D)** GSH content and **(E)** SOD activity were decreased in the HLF group compared with the LDH group. *p < 0.05, **p < 0.01, ***p < 0.001. Abbreviation: HLF: hypertrophy of ligamentum flavum; LDH: lumbar disc herniation; MRI: magnetic resonance imaging; ROS: reactive oxygen species; mtDNA: mitochondrial DNA; GSH: glutathione; SOD: superoxide dismutase.

The relative mtDNA copy number was significant lower in patients with HLF. The MDA content and ROS level were significantly increased in the HLF group, whereas the GSH content and SOD activity were markedly decreased in the HLF group (Figure 7).

### 3.8 Verification of hub genes expression

**FIGURE 8:**
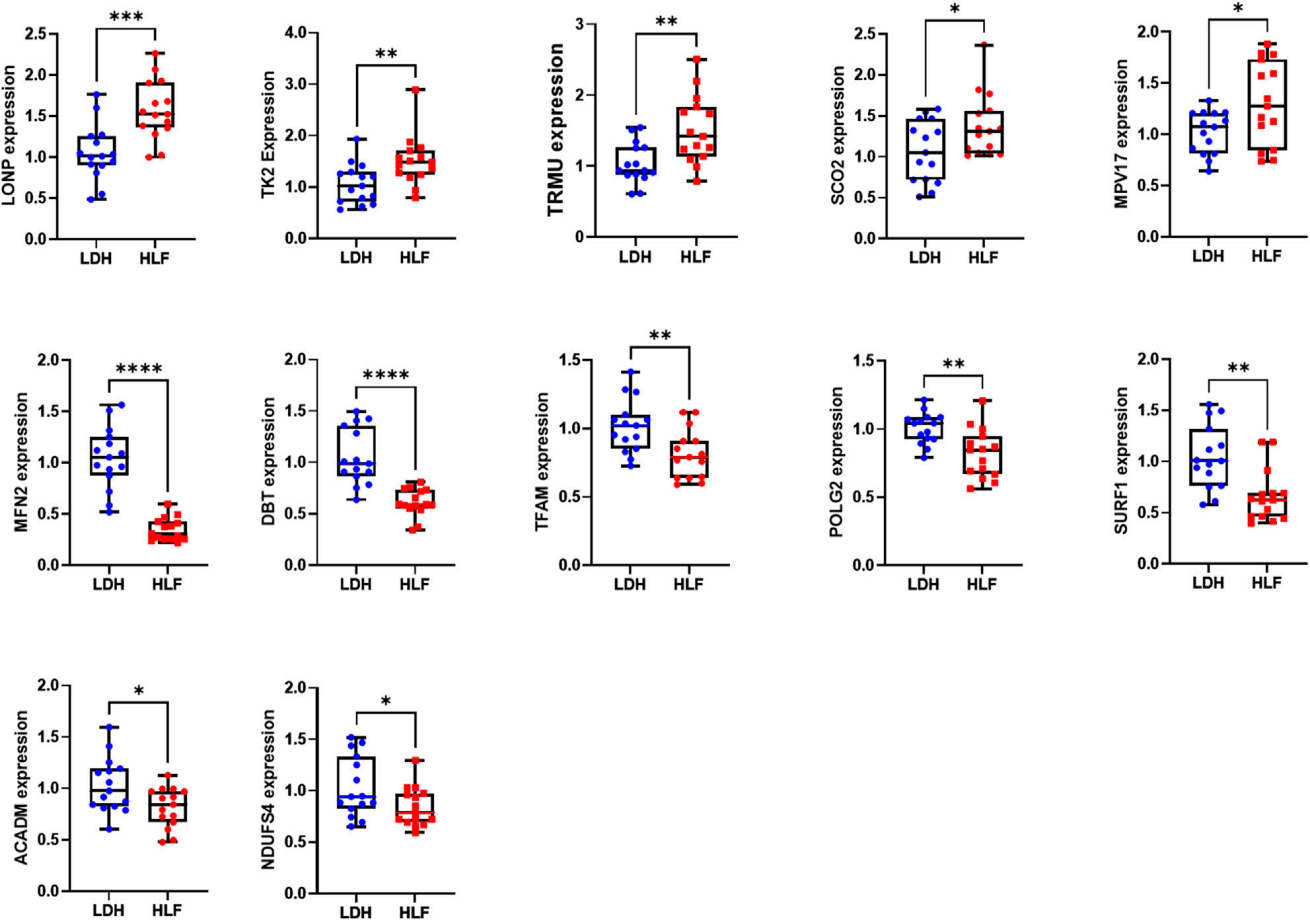
The relative mRNA expression of the hub genes. The relative mRNA expression level of LONP1, TK2, TRMU, SCO2, and MPV17 were significantly higher in HLF samples. The relative mRNA expression level of DBT, TFAM, MFN2, POLG2, SURF1, ACADM, and NDUFS4 were significantly lower in HLF samples. The difference in relative mRNA expression levels of CRAT, HADH, BCKDHB, NDUFV1, NDUFB9, ACAT1 HSD17B10, and NDUFAF4 between groups was not significant. *p < 0.05, **p < 0.01, ***p < 0.001, ****p < 0.0001. Abbreviation: HLF: hypertrophy of ligamentum flavum; LDH: lumbar disc herniation.

The expression of the above-mentioned 20 hub genes was verified by qPCR experiment. The relative mRNA expression level of LONP1, TK2, SCO2, TRMU, and MPV17 were significantly higher in HLF samples than in control samples, whereas the expression level of DBT, TFAM, MFN2, POLG2, SURF1, ACADM, NDUFS4 were significantly lower in HLF samples (Figure 8).

## 4 Discussion

The ligamentum flavum covers part of the posterior and lateral walls of the spinal canal and plays a role in maintaining the integrity of the spinal canal under normal circumstances. In most patients with LSS, the ligamentum flavum becomes hypertrophic and causes compression of the spinal nerve root or cauda equina, resulting in sensory and/or motor dysfunction in the lower extremities and/or perineum. If timely and correct treatment is not available, patients may face severe impairment of quality of life or even irreversible disability. Since there is currently no effective non-surgical treatment for reversing ligamentum flavum hypertrophy, it is very important to clarify its pathological mechanism and find suitable intervention targets. In recent years, several studies implied that oxidation stress and mitochondria dysfunction may involve in the development of HLF (Dechsupa et al., 2018; Chuang et al., 2020; Nagai et al., 2022). Mitochondria dysfunction may lead to abnormal mitochondrial morphology, decreased membrane potential, mitochondrial fusion/fission imbalance, increased reactive oxygen species (ROS) production, and induction of apoptosis, which have been proven to be responsible for aging and various age-related diseases (Zhang et al., 2022). However, the specific signaling pathways and intervention targets related to mitochondrial dysfunction in the process of HLF have not been demonstrated. Hence, in this study, we performed a bioinformatic analysis on an age-related HLF dataset (GSE113212) to identify hub genes and pathways associated with mitochondrial dysfunction in HLF.

The results of functional enrichment analysis suggested that the pathophysiological process of HLF is related to cellular oxidation and catabolic processes, while abnormalities in mitochondrial structure and function may play an important role. Clinically, patients with HLF and LSS are often complicated lipid metabolism-related diseases including peripheral arterial disease and aortic disease based on atherosclerosis (Uesugi et al., 2012; Beckworth et al., 2018). Recently, Sota et al. (Nagai et al., 2022) found a positive correlation between the expression of oxidized low-density lipoprotein (LDL) and the severity of HLF, while the increased oxidized LDL expression may affect HLF through the signaling of MAPKs. Chuang et al. (2020) demonstrated increased oxidative stress and inflammation in specimens of HLF and suggested oxidation could induce fibrosis in HLF, the process of which also involved the upregulation of NF-κB and iNOS and the intrinsic mitochondrial pathway of apoptosis. Overall, these results are consistent with our findings in this enrichment analysis.

In this study, we observed a significantly higher expression of ATPAF2, CLPB, CPOX, LONP1, MRPS34, PREPL, SCO2, SHMT2, TK2, TOMM40, and TXNRD2, and a lower expression of ABCB7, AGK, DBT, IBA57, MFN2, PDE2A, POLG2, and TFAM was observed in elderly individuals in the dataset. The genetic landscape showed that some of these genes are presented in close chromosomal locations, suggesting that these genes may be closely linked at the genome level and may have similar expression signatures at the transcriptome level. By analyzing the correlation between these genes, we found 7 pairs of them in positive correlations and 5 pairs in negative correlations. The linkage relationship between them can be regarded as the key to exploring the molecular interaction mechanism of HLF in the future.

In this study, 20 hub genes including NDUFV1, SURF1, NDUFS4, POLG2, LONP1, ACADM, TK2, HADH, MPV17, NDUFAF4, TFAM, MFN2, NDUFB9, ACAT1, CRAT, HSD17B10, DBT, BCKDHB, SCO2 and TRMU were identified. Most of these genes have been proven to have important roles in maintaining normal morphology and function of mitochondria (Chen et al., 2003; Pagniez-Mammeri et al., 2012; Rusecka et al., 2018). MFN2 is the gene that encodes mitofusin-2 (mfn-2), a protein that localizes to the outer mitochondrial membrane and is a key regulator of mitochondrial fusion, cell metabolism, autophagy, and apoptosis (Chen et al., 2003). Xu et al. (2020) found that MFN2 contributes to metabolic changes and inflammation during the aging of chondrocytes and osteoarthritis. In spinal structures, such as the intervertebral disc, Mfn-2 exerts an anti-apoptotic effect *via* ROS-dependent mitophagy and alleviates disc degeneration (Chen et al., 2020; Chuang et al., 2020). LONP1 encodes a mitochondrial matrix protein that belongs to the Lon family of ATP-dependent proteases. Recent studies (Xu et al., 2021; Guo et al., 2022; Xu et al., 2022) have demonstrated that abnormal expression of LONP1 was responsible for mitochondrial dysfunction-induced musculoskeletal disorders including skeletal muscle degeneration and idiopathic scoliosis. Mitochondrial transcriptional factor A (TFAM) was also proved to involve in chondrocyte degeneration. Wang et al. (2015) demonstrated that TFAM-mediated activation of the AMPK/SIRT-1/PGC-1α pathway can reduce mitochondrial biogenesis deficiency and procatabolic responses in osteoarthritis chondrocytes. The role of these hub genes in HLF has not yet been reported so far and may be considered targets for selective intervention in future studies.

The miRNAs and transcription factors of these hub genes were predicted using the miRNet database. Among these predicted miRNAs, some of them have been reported to be associated with the development of HLF. Hsa-miR-423-5p, hsa-miR-497-5p, has-miR-221-3p are miRNAs of MFN2. Mori et al. (2017) performed a microRNA transcriptome analysis on hypertrophy of ligamentum flavum and found deregulation of hsa-miR-423-5p and hsa-miR-497-5p were closely correlated to the severity of HLF. Xu et al. (2016b) found that downregulation of hsa-miR-221 might contribute to LF hypertrophy by promoting collagens I and III expression. Hsa-miR-21 is the miRNA of SCO2. Sun et al. (2020) demonstrated that the over-expression of miR-21 was associated with the IL-6 expression, and promotes inflammation and fibrosis in ligamentum flavum.

The TF-gene network showed that SP1, PPARGC1A, YY1, MYC, PPARG, and STAT1 play important roles in HLF. Disrupted expression of SP1 was demonstrated to be responsible for abnormal expression of mitochondrial complex I genes, NDUFV1 and NDUFV2 (Ben-Shachar and Karry, 2007). Experiments have shown that Sp1 (Specificity protein-1) is involved in the expression of catabolic enzymes in the inflammatory factors-induced degeneration of the nucleus pulposus and chondrocytes (Sylvester et al., 2013; Xu et al., 2016c). PPARGC1A (peroxisome proliferator-activated receptor gamma coactivator-1) and its target MFN2 have been proven both involved in mitochondrial biogenesis and the maintenance of the mitochondrial network (Gastaldi et al., 2007). In the process of intervertebral disc degeneration, PPARGC1A has been proven to protect annulus fibrosus cells against apoptosis under oxidative stress (Xu et al., 2019). YY1 (Yin Yang 1) is a ubiquitously distributed transcription factor belonging to the GLI-Kruppel class of zinc finger proteins (Gaston and Fried, 1994). Studies have demonstrated that YY1 is involved in the regulation of the bi-directional promoter of the SURF1 (Gaston and Fried, 1994; Gaston and Fried, 1995). However, the role of these transcription factors in HLF needs further study.

The role of immune infiltration in HLF has been demonstrated in recent years. Chuang et al. (2020) found that inflammation was involved in HLF and was associated with oxidative stress. Saito et al. (2017) revealed that the gene expression of collagen markedly increased in the ligamentum flavum cells with infiltrating macrophages, suggesting that macrophage infiltration was crucial for HLF by stimulating collagen production. In our study, an increase in immune cell infiltration, especially macrophages infiltration was demonstrated in samples of age-related HLF, which was consistent with existing researches. Meanwhile, hub genes including ACADM, BCKDHB, HSD17B10, MFN2, NDUFAF4, and LONP1 were found to have a close correlation with the infiltration level of immune cells, suggesting that the genes related to mitochondrial dysfunction may involve in the immune infiltration process in HLF.

In conclusion, this study revealed the mitochondrial dysfunction-related key genes, regulatory pathways, TFs and miRNAs underlying the development of HLF, which improved the understanding of molecular mechanisms and the development of novel therapeutic targets for HLF.

## Data Availability

The original contributions presented in the study are included in the article/supplementary material, further inquiries can be directed to the corresponding authors.
